# Can artificial intelligence pass the test? Evaluating chatbot scores on pediatric gastroenterology board‐style questions

**DOI:** 10.1002/jpr3.70177

**Published:** 2026-04-07

**Authors:** Sharmilaa Babu, Arun Ajmera

**Affiliations:** ^1^ Department of Pediatric Gastroenterology, Hepatology, and Nutrition Duke University School of Medicine Durham North Carolina USA

**Keywords:** clinical reasoning, digital health tools, large language models, medical education

We appreciate Dr. Weizman's interest in our study evaluating chatbot performance on pediatric gastroenterology board‐style questions and welcome the chance to clarify a few points.^1^, ^2^

We focused on text‐based performance because that's still how most clinicians and trainees use these tools. When we extracted the Pediatric Review and Education Program Gastroenterology (PREP GI) material, the image files weren't available in a usable form. Rather than trying to recreate or approximate them, we analyzed the questions as they could be exported to reflect real world use, not idealized peak performance.^3^, ^4^

For model selection, we compared platforms that clinicians could access without specialized infrastructure. At the time, several high performing systems (e.g., Claude, Gemini, and MedPaLM) were restricted or not available through institutional accounts, and others raised reproducibility concerns tied to proprietary access. Our goal wasn't to test every model, but to evaluate tools trainees and pediatric gastroenterologists could readily use.

We agree that prompting can affect accuracy. Still, we chose minimal prompting to better reflect how people pose questions in routine clinical settings; adding engineered prompts risked overstating real‐world performance.^5^, ^6^ While recognizing statistical power limitations, each PREP item was tested once as repeated testing introduces learning effects where large language models internalize prior question answer pairs.

Finally, our domain level analyses followed the structure and distribution of the PREP GI categories. As some sections have only a small number of questions, results were treated descriptively rather than inferentially to avoid overinterpreting limited data.

We value the dialogue and share the goal of strengthening future evaluations as access to multimodal models grows.

## CONFLICT OF INTEREST STATEMENT

The authors declare no conflicts of interest.
